# Assessment of the Dietary Intake and Nutritional Status of Polish Professional Futsal Players: A Descriptive Study—Do Futsal Players Require Nutritional Education?

**DOI:** 10.3390/nu15173720

**Published:** 2023-08-25

**Authors:** Anna Gogojewicz, Anna Straburzyńska-Lupa, Tomasz Podgórski, Paulina Frajtag, Karol Bibrowicz, Ewa Śliwicka

**Affiliations:** 1 Department of Food and Nutrition, Poznan University of Physical Education, 61-871 Poznań, Poland; gogojewicz@awf.poznan.pl; 2 Department of Physical Therapy and Sports Recovery, Poznan University of Physical Education, 61-871 Poznań, Poland; straburzynskalupa@awf.poznan.pl (A.S.-L.); frajtag@awf.poznan.pl (P.F.); 3 Department of Physiology and Biochemistry, Poznan University of Physical Education, 61-871 Poznań, Poland; podgorski@awf.poznan.pl; 4 Department Science and Research Center of Body Posture, Kazimiera Milanowska College of Education and Therapy, 61-473 Poznań, Poland; bibrowicz@wp.pl

**Keywords:** sports nutrition, athletes, nutritional status

## Abstract

Futsal is a discipline with high training and nutritional requirements. Despite growing research interest in athletes’ diet and nutritional status, no studies have examined Polish male futsal players. Therefore, the aim of this descriptive study was an assessment of the dietary intake and nutritional status in a selected group of futsal players. The study comprised 11 members of a top Polish futsal team (aged 26 ± 3.62 years). Dietary intake was assessed using a standardized 3-day food record. Body composition, total energy expenditure, physical fitness level, and concentrations of the biochemical indices of each participant were estimated. The energy availability in the diet was lower than recommended. Moreover, low consumption of carbohydrates was stated, as well as an inadequate intake of Vitamins E and D. Higher protein and cholesterol intake than recommended were also observed. To conclude, our results point to the need for educating athletes and coaches, particularly teaching about proper food choices, promoting quality foods, and, in some cases, using individual dietary plans to meet energy and nutrient needs. Nutrition education would help to improve their dietary and health habits and optimize their performance in sports training.

## 1. Introduction

Futsal is an indoor variation of soccer that has been FIFA-sanctioned since 1988 [1]. In official competitions, futsal is a five-a-side game (one goalkeeper and four field players, commonly named the defender, the right and left wingers, and the pivot) on smaller pitches compared with field sports, and with unlimited substitutions [2,3,4,5]. This physically demanding game requires strong tactical and technical skills, with a large amount of sprinting and more phases of high intensity than other intermittent sports [2]. Futsal is therefore defined as a high-intensity intermittent sport [6], in which there can be significant demands on the aerobic and anaerobic metabolism [2,7]. Research has shown that professional futsal requires players to work at an intensity of or above 60 mL·kg^−1^·min^−1^ [3]. 

Taking all this into account, futsal players have high nutritional requirements in terms of both energy expenditure and fluid and electrolyte loss during training and matches [8]. An effective nutritional strategy between training sessions is also crucial for the effectiveness of the recovery process and adaptation to different fatigue mechanisms [9,10]. Proper nutrition, considering the quality and quantity of food consumed at appropriate times (so-called nutrient timing), is fundamental for body building and optimizing sports performance, health, and well-being [9,11,12]. In doing so, a number of sport-specific factors, body characteristics, or tasks specific to the sporting position must be considered [12]. Nutritional faults due to dietary imbalances and energy deficiencies can contribute to a number of physical problems, such as a loss of lean body mass, incomplete recovery, endocrine disorders, increased resting heart rate, and psychological issues such as apathy or reduced concentration [13,14,15].

The nutritional recommendations published by international sports organizations, such as the International Olympic Committee (IOC), the American College of Sports Medicine (ACSM), and the International Society of Sports Nutrition (ISSN), are therefore important, primarily to support the physical demands of training and optimize performance, improve recovery, and support the nutritionists working with athletes to meet individual nutritional needs [12]. However, reviewing the available literature does not provide specific nutritional recommendations for futsal players. 

Recently, there has also been growing interest in the role of Vitamin D in the human body [16,17,18], especially in athletes [19,20,21,22]. Ongoing research has shown that low Vitamin D levels have a negative impact on muscle strength, power, and endurance, and increase musculoskeletal injuries and inflammation [23]. Therefore, the high prevalence of Vitamin D deficiency in athletes [24] indicates the need to monitor its levels [25,26], which seems particularly relevant in indoor athletes such as futsal players [27].

In recent years, body composition analysis has become part of athletes’ assessments as an important determinant of health and performance [28]. Different strategies are used to ensure the optimal body composition for each athlete and sport. At the same time, it has been pointed out that research is needed to establish reference data for the body composition indices of athletes in different sports [28].

Despite growing research interest in athletes’ diet and nutritional status, no studies have examined Polish male futsal players. Therefore, the present study aimed to investigate the diet and nutritional status of professional futsal players.

## 2. Materials and Methods

### 2.1. Participants

Initially, the study group consisted of 12 men, who were members of a top Polish futsal team. Due to a problem with blood collection, the number of athletes participating in the research decreased to 11 men. The mean age of athletes was 26 ± 3.62 years, and the average training experience was 7 ± 2.38 years. All subjects were free from injury and illness, and declared that they had not introduced any changes in their lifestyles, training elements, nutrition, and/or supplementation. The characteristics of the participants are presented in Table 1.

The study protocol was reviewed and approved by the Bioethics Committee of the Poznan University of Medical Sciences (reference number 518/21, with the annex number 564/23). Participation in the study was voluntary; athletes were informed about the purpose of the study and could withdraw at any time. All study participants gave written informed consent. All procedures were carried out in accordance with the ethical standards of the Helsinki Declaration of 1975.

### 2.2. Study Design

All procedures were conducted at the end of the preparatory period (September 2021), which lasted six weeks.

### 2.3. Anthropometric and Body Composition Measurements

All anthropometric measurements were conducted in the morning, in a fasting state, by the same specialist (a certified nutritionist). Body mass and height were measured using a certified digital medical-grade scale and a mechanical measuring rod (WPT 60/150.O, Radwag, Radom, Poland) with an accuracy of 0.1 kg for weight and 0.5 cm for height, respectively. Body composition was assessed by the bioimpedance method, using the TANITA BC-420 analyzer (Tokyo, Japan) with GMON Professional software version 3.4.2 (Medizin & Service GmbH, Chemnitz, Germany). Body composition was measured strictly following the recommended measurement conditions [29], as described previously [30,31]. The athletes were also asked to pay attention to proper hydration because inadequate hydration due to excessive fluid loss or improper fluid intake can result in unreliable results of body composition analyses by bioelectrical impedance analysis (BIA).

### 2.4. Nutritional Assessment

The assessment of dietary intake was conducted using a standardized 3-day food record. The participants were asked to express the size of their meals in common measurement units (e.g., glasses, cups, bowls, spoons, etc.). The information thus obtained was adjusted using the album of photographs of food products and dishes elaborated by the National Food and Nutrition Institute in Warsaw (Poland) [32]. Quantitative analysis of the composition of daily food rations was performed using the Nuvero online application (NUVERO-VOIX, Koszalin, Poland; access date: October 2022), which uses a database developed by the National Food and Nutrition Institute in Warsaw [33]. The mean intakes of energy and nutrients were compared with recommendations of the International Society of Sports Nutrition (ISSN) [14] and the American College of Sports Medicine [34].

### 2.5. Exercise Energy Expenditure

Exercise energy expenditure (EEE) was assessed over three days, in which athletes collected their dietary data. EEE was measured during team training sessions, which included futsal practice and control matches. During the training, the Polar Team2 System with software version 1.4 (Polar Electro, Kampele, Finland) was used to record the players’ heart rate. Energy expenditure was calculated using proprietary algorithms.

### 2.6. Energy Availability

Energy availability (EA) is defined as the amount of dietary energy remaining after exercise training for all other metabolic processes, normalized to fat-free mass (FFM) (or lean body mass) [35]. The conventional EA equation is as follows:

EA (kcal·kg·FFM^−1^·day^−1^) = [EI (kcal·day^−1^) − EEE (kcal·day^−1^)]·FFM (kg)^−1^

where EI is the energy intake and EEE represents exercise energy expenditure.

### 2.7. Exercise Tests and Physical Fitness Estimation

The futsal players performed the Yo-Yo Intermittent Recovery Test Level 2 (YYIR2) according to established procedures [36]. The test was conducted indoors on a wooden sports floor and consisted of 2 × 20 m shuttle runs at progressively increasing speeds (initial speed: 13 km/h) with a 10 s active recovery period between runs. The test was terminated when the subjects twice failed to reach the starting line or the participant could not complete another shuttle at the dictated speed. After completing the test, maximal oxygen consumption (VO_2max_) was calculated using the following formula [36]:

VO_2max_ (mL·min^−1^·kg^−1^) = (YYIR2 distance (m) · 0.0136) + 45.3

### 2.8. Biochemical Analyses

Blood samples for biochemical analyses were obtained from the antecubital vein under fasting conditions (between 08:00 AM and 10:00 AM). The samples were collected following all safety standards into serum-separating tubes (9 mL, S-Monovette, SARSTEDT, Nümbrecht, Germany) and centrifuged at 2000× *g* for 10 min at 4 °C. The serum was separated from the sample and stored at −70 °C.

In the serum, the total cholesterol (Cat. No. 7-204), high-density lipoprotein (HDL; Cat. No. 7-279), low-density lipoprotein (LDL; Cat. No. 7-280), triglycerides (Cat. No. 7-253), total protein (Cat. No. 7-236), albumin (Cat. No. 7-238), and glucose (Cat. No. 7-201) concentrations were analyzed. All measurements were performed using the Accent 220S automatic biochemical analyzer (Cormay, Łomianki, Poland) and PZ Cormay S.A. Company reagents (Łomianki, Poland). The serum concentration of 25-hydroxyvitamin D was determined using an IDS EIA kit (IDS, Boldon, UK; Cat. No. AC-57SF1).

### 2.9. Statistical Analyses

All analyses were performed using the Statistica 13.0 software package (StatSoft, Tulsa, OK, USA). Data are presented as the means, standard deviations (SD), medians (Me), quartiles (Q_1_–Q_3_), and minimum–maximum (Min–Max). The Shapiro–Wilk test was used to check the data for a normal distribution. The level of statistical significance was set at *p* < 0.05. Spearman’s rank analysis was used to calculate the correlation coefficients.

## 3. Results

### 3.1. Anthropometry, Body Composition, and Physical Fitness

The basic characteristics of the examined players are presented in Table 1. They were lean athletes with relatively high mean values of FFM and a VO_2max_ of 59.7 ± 2.07 (mL·min^−1^·kg^−1^).

### 3.2. Biochemical Indices of Nutritional Status

As shown in Table 2, the mean values of the biochemical indicators of nutritional status were within the reference values.

### 3.3. Nutritional Evaluation

The average energy value of the diet and the intake of selected nutrients in futsal players are presented in Table 3. The obtained nutritional results were compared with recommendations for athletes with similar physical effort: 2–3 h per day of intense exercise performed five or six times per week and high-volume intense training (3–6 h per day of intense training in one or two workouts for 5–6 days per week) [14]. The diet of the investigated players was characterized by low energy availability (LEA), although the average energy intake was 2706.63 ± 442.12 kcal·day^−1^ and met the requirements for this sporting discipline. However, an imbalance in the intake of macronutrients was found. The futsal players’ diet was characterized by a lower than recommended content of carbohydrates and a slightly higher protein content. Higher cholesterol intake than recommended was also observed. The consumption of fatty acids was sufficient, except for saturated fatty acids (share in total energy), which exceeded the recommended standards.

The athletes consumed too high an intake of sodium and potassium. The average daily food rations were also characterized by a high intake of calcium, phosphorus, magnesium, iron, zinc, Vitamin A, Vitamin C, Vitamin B1, Vitamin B6, and Vitamin B12. On the other hand, values lower than recommended were noted in the consumption of Vitamins D and E (Table 4).

### 3.4. Correlations

**Figure 1:**
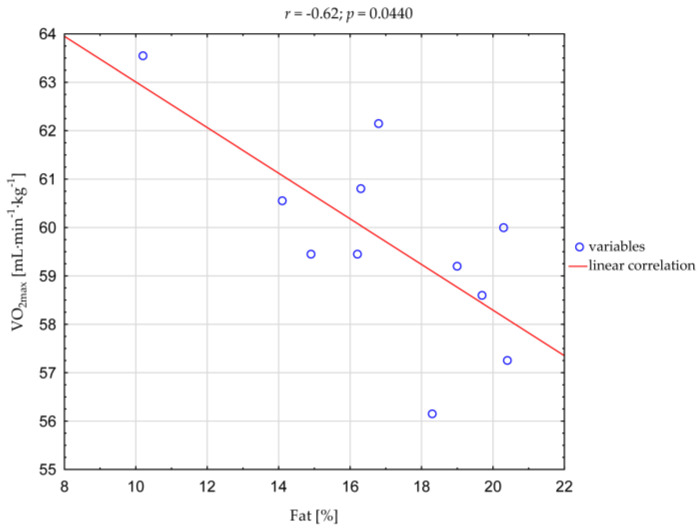
Correlation between VO_2max_ and percentage of fat content in the futsal players (*n* = 11).

The correlation analysis showed a negative association between VO_2max_ and the percentage of fat content (*r* = −0.62; *p* = 0.0440; Figure 1).

## 4. Discussion

Over the last few decades, compelling evidence has highlighted the importance of athletes’ nutritional strategies for optimizing sports performance and enhancing recovery [14]. Hence, there has been a growing interest among researchers in the diet and nutritional status of athletes in various sports. In the available literature, only a few studies have assessed the nutritional status of futsal players, and, to our knowledge, this is the first study reporting on the diet and nutritional status of Polish professional male futsal players.

Given the nature of the game, it is crucial to ensure sufficient calories to balance energy expenditure to optimize performance through nutrition [14], and to maintain immune and metabolic functions [37]. Due to the high-intensity effort, the nutritional requirements for futsal players appear to be similar to other indoor team sports such as handball, basketball, and volleyball [14,38]. Energy expenditure is related to the typical specific actions of futsal (e.g., acceleration, deceleration, sprinting, changes of direction, and jumping [5]) and several other factors, such as the body type of the players and the position occupied during the game [1,39].

The present study showed that the diet of the investigated players was characterized by low energy availability (LEA) and insufficient amounts of carbohydrate compared with their estimated requirements. Although the average energy intake of the athletes studied was in line with sports associations’ recommendations, the energy provided in the study group’s usual rations varied and was sometimes insufficient to meet the daily energy requirements. More than 2500 kcal, the minimum recommended by the ACSM [34], was provided in the diets of only 45% of the studied athletes. The lower total energy intake and LEA can be explained by the lower intake of carbohydrates because, in our athletes, we also found that the carbohydrate intake was lower than recommended, with an average of 4.1 g·kg_bw_^−1^, and 45% of subjects consumed less than 4 g·kg_bw_^−1^. Similarly, in a study by Kagawa et al. [11], Japanese futsal players consumed insufficient amounts of energy and nutrients relative to their estimated requirements. In addition, Jenner et al. [12], in their systematic review, indicated that this phenomenon is common in team sports.

Given that the majority of available studies indicate that carbohydrates are the preferred macronutrient in the diets of team game players [40], the low supply of carbohydrates (CHO) in the subjects’ daily rations is a concern. Athletes use their CHO intake to improve sporting performance by providing a fuel substrate and enhancing the bioavailability of other supplements [40,41]. High CHO availability before and during exercise is essential, as glycogen depletion is a limiting factor for performance during high-intensity exercise sessions [40,42]. Insufficient CHO intake may impair athletic performance and result in the degradation of muscle tissues due to utilizing amino acids in energy metabolism [11]. At the same time, recovery may be inadequate, as muscle glycogen stores are unlikely to adequately replenish by the next training session [10,37]. This may also lead to an increased risk of chronic fatigue or injury [14,43]. Therefore, increasing the subjects’ total dietary energy intake seems beneficial by increasing the intake of carbohydrate-rich foods (e.g., cereals, rice, potatoes, and fruits). Moreover, it should be noted that CHO timing is crucial for optimal fueling [9]. In addition to being an energy substrate, glycogen is now considered a regulator of the pathways regulating exercise adaptations [44,45]. Low CHO availability promotes endurance adaptations such as mitochondrial biogenesis and increased lipolysis. Considering all this, the diet should be periodically adjusted according to the training load and training goals [42,46].

Our subjects did not consume adequate amounts of energy and carbohydrates, and the intake of other macronutrients in their diets exceeded the current recommended daily intake, which could indicate that athletes did not make quality nutritional choices.

In our study, a slightly higher protein intake than recommended [14,47] was observed. A detailed analysis showed that athletes are more likely choose readily available animal products, such as meat, cheese, and eggs. They were less likely to choose plant products from the legume group. This seems to call for increased awareness of the principles of proper nutrition in this group of athletes, given that the study was conducted in September when fresh vegetables are most readily available in Poland.

However, despite the overall proportion of fats in the diet of the studied athletes (32.7%) being in line with the recommendations, only one of the athletes (9%) had an acceptable cholesterol intake. In contrast, the intake of saturated fatty acids was too high in 100% of cases. This fact is explained by the increased intake of meat products and eggs in the daily rations. Parallel biochemical blood tests showed that some athletes had too high total cholesterol and triglyceride levels (9% and 18%, respectively), and others (27%) had total cholesterol levels close to the upper limit of the reference level.

The diet of our athletes was characterized by an insufficient intake of Vitamin E. This may be related to a lack of knowledge, as September, the month of the study, is rich in tocopherol-rich fruits and vegetables such as tomatoes, broccoli, brussels sprouts, spinach, peaches, and blackcurrants. Respondents should also include olive oil, sunflower oil, almonds, hazelnuts, sprouts, and cereal seed germ in their diets.

Although the dietary intake of Vitamin D was deficient, the serum levels of all tested subjects were high. Vitamin D is synthesized in the skin and produced in the body following exposure to Ultraviolet B (UVB) radiation from the sun [26] or from nutritional sources, such as fatty fish, egg yolk, dairy products, and fortified foods, where Vitamin D can be obtained [26]. Given that in Poland, the solar angle and weather conditions suitable for Vitamin D synthesis occur between late April and early September [48], this may confirm the significant contribution of sun exposure to Vitamin D synthesis during the preparation period. In contrast, 80% of outdoor athletes and 84% of indoor athletes in winter and 42% and 83% in summer were found to have inadequate Vitamin D levels in the study by Krzywański et al. [27]. Their results and ours confirm that indoor athletes need wintertime Vitamin D supplementation.

It is very likely that an inadequate intake of fruit and vegetables, with a predominance of processed products high in salt, also led to excess sodium in the athletes’ diets. Given the high losses of this component in sweat, these values are not worrying. However, from a practical point of view, the observed high potassium intake could be considered significant, as an imbalance in electrolyte concentrations (Na^+^ and K^+^) can lead to painful, sudden, and involuntary skeletal muscle contractions during or after training [49].

Morphology, body composition, and posture are essential in different sports disciplines [1]. There are a number of techniques used to measure body composition. They include laboratory (e.g., dual-energy x-ray absorptiometry (DXA), hydrodensitometry, air displacement plethysmography) and field methods (e.g., single and multi-frequency bioelectrical impedance analysis (BIA), skinfold measurements) [50,51]. Although DXA is considered the “gold standard” of body composition measurements, it is expensive and requires highly specialized personnel, so field methods are often used in sports practice [52]. In our study, we used the BIA method, which allowed us to estimate parameters such as FM, FFM, and TBW through the algorithm utilizing regression formulas based on weight, height, age, sex, and whole-body resistance [53,54]. Moreover, a wide range of predictive equations for specific populations, such as athletes involved in different sports, is available in the literature [53,54]. Unfortunately, our research did not measure skinfolds, which are necessary for using the new formula proposed by Giro et al. [52] for futsal players.

Several researchers have suggested that anthropometric characteristics may be related to components of fitness in team sports [55,56,57,58] and are associated with the obtained results [59]. The relationship between physical fitness and body composition confirmed the negative correlation obtained in our research between VO_2max_ and fat content (Figure 1). Our results are in line with those obtained by other authors [60,61,62,63].

Differences have also been found between futsal players regarding their playing position [58], which was confirmed in our study. Similar to the results of other authors [64,65], the pivots and goalkeepers investigated in our study had a higher percentage fat content and BMI values than the other players. The main characteristics of the pivot position in futsal are physical strength and good use of the body and the spinning technique to protect the ball. However, it is essential to note that excess body weight may reflect increased fat-free mass (FFM) and not necessarily increased body fat (FM). In the case of our pivots, we found high body fat levels in athletes with 6 and 8 years of training experience (19% and 20.4%, respectively) but also quite high levels of FFM (64.2 kg and 72.6 kg, respectively). Notably, regardless of body composition, excess weight is an additional burden during most training and match activities and may be associated with increased fatigue, injury risk [66], and reduced performance-related ability. Thus, optimizing body mass and the correct body composition of players should be considered as a training and nutritional goal to improve sporting performance [67].

Our study has some potential limitations that should be noted. The first is the relatively small sample size. However, this is due to the fact that the study included only one team of Polish professional futsal players. The second is the lack of methods considered as the “gold standard” for assessing body composition, such as magnetic resonance for quantifying muscle mass or DXA for fat mass. We used one of the field methods (BIA), which is simple, quick, and non-invasive. However, it should be noted that the obtained results depend on BIA-based predictive equations. The study was conducted once, in September, when Polish seasonal fruit and vegetables were available. Repeating this research at other times of the year would be interesting. With these limitations in mind, this study provides information on professional Polish futsal players’ diet and nutritional status, drawing attention to the need for effective strategies in this area.

## 5. Conclusions

To conclude, our results point to the need to educate athletes and coaches, particularly teaching about proper food choices, the promotion of quality foods, and, in some cases, the use of individual dietary plans to meet energy and nutrient needs. Nutrition education would help to improve their dietary and health habits and optimize their performance in sports training.

## Figures and Tables

**Table 1:** Demographic and anthropometric data of the futsal players (*n* = 11).

Variables	Mean ± SD	Me (Q_1_–Q_3_)
Age (years)	26 ± 3.62	26 (22.0–28.0)
Body height (cm)	179.8 ± 6.81	178.0 (174.0–187.0)
Body mass (kg)	79.3 ± 8.49	79.2 (72.7–85.5)
Fat (%)	16.9 ± 3.09	16.8 (14.9–19.7)
FM (kg)	13.6 ±3.50	12.9 (12.1–15.6)
FFM (kg)	65.7 ± 5.67	64.2 (61.0–69.9)
TBW (%)	57.4 ± 2.98	57.5 (55.1–58.6)
BMI	24.5 ± 1.40	24.9 (23.5–25.3)
Training experience (years)	7 ± 2.38	6.0 (5.0–9.0)
VO_2max_ (mL·min^−1^·kg^−1^)	59.7 ± 2.07	59.5 (58.6–60.8)

Values are expressed as means ± SD and Me (Q_1_–Q_3_). BMI, body mass index; FM, fat mass; FFM, fat-free body mass; TBW, total body water.

**Table 2:** Concentrations of selected biochemical indices in the futsal players (*n* = 11).

Variables	Mean ± SD	Me (Q_1_–Q_3_)
Glucose (mg·dL^−1^)	92.6 ± 5.95	93.0 (91.5–95.5)
Protein (g·dL^−1^)	7.6 ± 0.23	7.6 (7.5–7.9)
Albumin (g·dL^−1^)	4.8 ± 0.17	4.8 (4.7–4.8)
Total cholesterol (mg·dL^−1^)	170.4 ± 20.14	164.5 (153.5–186.5)
HDL-CH (mg·dL^−1^)	56.9 ± 7.93	57.9 (50.2–61.4)
LDL-CH (mg·dL^−1^)	99.4 ± 18.12	100.5 (83.1–109.2)
Triglycerides (mg·dL^−1^)	96.4 ± 35.96	84.50 (70.5–108.5)
25(OH)D (ng·dL^−1^)	59.2 ± 8.86	60.1 (51.4–69.0)

Values are expressed as means ± SD and Me (Q_1_–Q_3_). HDL-CH, high-density lipoprotein cholesterol; LDL-CH, low-density lipoprotein cholesterol.

**Table 3:** Energy intake, energy expenditure, energy availability, and intake of selected nutrients in the futsal players (*n* = 11).

Nutrient	Mean ± SD	Me (Min–Max)	ISSN	ACSM
Energy intake (kcal)	2706.6 ± 442.12	2490.6 (2006.0–3724.1)	2000–7000	2500–8000
Energy expenditure during exercise (kcal)	1029.6 ± 110.32	966.3 (911.5–189.3)		
Energy availability (kcal·kg FFM^−1^)	25.7 ± 6.35	23.6 (18.1–41.6)	>45	
Fluids (mL)	2721.6 ± 986.93	2607.4 (1358.5–4311.7)	2500 *	
CHO				
g	321.8 ± 78.33	306.2 (235.9–490.4)	250–1200 g/day for 50–150 kg	
% energy	47.7 ± 5.44	50.2 (38.5–52.7)	55	
g·kg_bm_^−1^	4.1 ± 1.24	4.0 (2.8–7.2)	5–8	6–10
Fiber (g)	25.2 ± 5.27	25.7 (17.4–34.8)	25 g	
PRO				
g	141.8 ± 19.2	147.5 (102.9–167.4)	60–300 g/day for 50–150 kg	
% energy	21.2 ± 2.97	21.9 (17.5–25.5)	20	
g·kg_bm_^−1^	1.8 ± 0.37	1.8 (1.24–2.47)	1.4–1.8	1.5–2.0
Vegetable proteins (g)	33.8 ± 10.47	31.1 (20.3–50.9)		
Animal proteins (g)	97.5 ± 18.1	93.1 (76.1–130.8)		
FAT				
g	98.1 ± 17.99	91.2 (75.0–128.6)		
% energy	32.7 ± 3.93	31.3 (27.1–40.0)	30–35	20–35
g·kg_bm_^−1^	1.3 ± 0.31	1.2 (0.94–1.86)	0.5–1.0	0.5–1.5
Cholesterol (mg)	516.0 ± 217.67	470.8 (225.2–933.0)	<300	
SFA (g) SFA (%)	38.0 ± 9.39 12.5 ± 1.31	33.6 (28.8–61.0) 12.3 (9.7–14.7)		<10
PUFA (g) PUFA (%)	13.6 ± 3.12 4.6 ± 1.09	13.9 (8.3–18.5) 4.6 (3.0–6.9)	0.5–1.0 ^§^ 6–10	
MUFA (g) MUFA (%)	36.3 ± 8.23 12.1 ± 2.17	34.9 (25.3–52.8) 11.6 (9.8–16.4)		
Omega-3FA (g) Omega-3FA (%)	2.9 ± 0.87 1.0 ± 0.33	2.7 (1.6–4.7) 1.0 (0.6–1.5)	1–2	
Omega-6 FA (g) Omega-6FA (%)	10.6 ± 2.62 3.5 ± 0.85	11.6 (6.7–14.0) 3.4 (2.4–5.5)	5–8	

Values are expressed as the means ± SD and Me (Min–Max). CHO, carbohydrates; PRO, proteins; Fat, lipids; SFA, saturated fatty acids; PUFA, polyunsaturated fatty acids; MUFA, monounsaturated fatty acids, * European Food Safety Authority; ^§^ American Heart Association, American Dietetic of Canada.

**Table 4:** Daily dietary intake of vitamins and minerals in the futsal players (*n* = 11).

Vitamins and Minerals	Mean ± SD	Me (Min–Max)	ISSN	ACSM
Sodium (mg)	2913.3 ± 962.60	2666.1 (1239.5–4237.1)	500 *	340
Potassium (mg)	4418.3 ± 804.49	4581.40 (2566.1–5628.2)	2000 *	
Calcium (mg)	1197.7 ± 444.98	1283.1 (643.2–1993.2)	1000	1500
Phosphorus (mg) (phosphate salts)	1986.3 ± 301.11	2000.4 (1574.6–2669.4)	700	
Magnesium (mg)	447.6 ± 74.89	453.5 (335.3–560.3)	420	
Iron (mg)	14.1 ± 2.51	13.9 (9.3–18.3)	8 (ages 19–50)	6 mg/1000 kcal
Zinc (mg)	14.4 ± 2.79	14.8 (91.0–20.2)	11	
Vitamin A (μg)	1158.9 ± 271.93	1235.9 (667.2–1478.0)	900 μg/d	
Vitamin D (μg)	3.2 ± 2.40	2.3 (0.6–8.5)	5 (age < 51)	1500–2000 IU
Vitamin E (mg)	13.0 ± 3.14	13.2 (8.2–17.4)	15	
Vitamin C (mg)	125.0 ± 55.45	130.7 (45.5–204.8)	90	
Vitamin B1 (mg)	1.6 ± 0.32	1.6 (1.2–2.3)	1.2	
Vitamin B6 (mg)	3.1 ± 0.65	3.1 (1.6–3.9)	1.3 (age < 51)	
Vitamin B12 (μg)	5.5 ± 2.29	5.0 (2.5–8.8)	2.4	

Values are expressed as the means ± SD and Me (Min–Max). Recommended dietary allowances (RDA) were based on the recommendations of the 2002 Food and Nutrition Board of the National Academy of Sciences—National Research Council. * Estimated minimum requirement.

## Data Availability

The data supporting reported results are available on request from the corresponding author (E.Ś.).
